# Green synthesis of a Cu/MgO nanocomposite by *Cassytha filiformis* L. extract and investigation of its catalytic activity in the reduction of methylene blue, congo red and nitro compounds in aqueous media

**DOI:** 10.1039/c7ra13491f

**Published:** 2018-01-18

**Authors:** Mahmoud Nasrollahzadeh, Zahra Issaabadi, S. Mohammad Sajadi

**Affiliations:** Department of Chemistry, Faculty of Science, University of Qom Qom 3716146611 Iran mahmoudnasr81@gmail.com; Center of Environmental Researches, University of Qom Qom Iran; Scientific Research Center, Soran University PO Box 624, Kurdistan Regional Government Soran Iraq; Department of Pharmacy, Rwandz Private Technical Institute Kurdistan Regional Government Rwandz Iraq

## Abstract

This work reports the green synthesis of a Cu/MgO nanocomposite using *Cassytha filiformis* L. extract as a reducing agent without stabilizers or surfactants. The immobilization of Cu NPs was confirmed by Fourier transform infrared spectroscopy (FT-IR), X-ray diffraction (XRD), transmission electron microscopy (TEM), field emission scanning electron microscopy (FESEM) and energy dispersive X-ray spectroscopy (EDS). The Cu/MgO nanocomposite acts as a heterogeneous and recyclable catalyst for the reduction of 4-nitrophenol (4-NP), 2,4-dinitrophenylhydrazine (2,4-DNPH), methylene blue (MB) and congo red (CR) using sodium borohydride in aqueous media at room temperature. The catalyst was recycled multiple times without any significant loss of its catalytic activity.

## Abstract 2

This work reports the green synthesis of a Cu/MgO nanocomposite using *Cassytha filiformis* L. extract as a reducing agent without stabilizers or surfactants.

## Graphical abstract

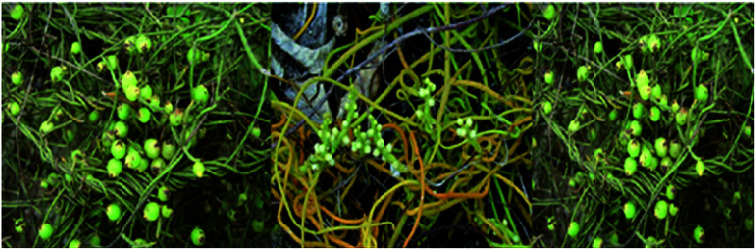

## Introduction

The presence of azo dyes and nitroarene compounds in waste waters is of great concern for researchers. The dyes and nitroarene compounds used in various industries are often highly toxic to aquatic organisms because these are biologically and chemically stable. Therefore, it is arduous to remove them by natural degradation processes.^1,2^ Degradation of the dyes to nondangerous products^3–5^ and also nitroarenes to useful compounds^6,7^ are very important reactions. Recently, scientists have introduced a reduction process in the presence of metal nanoparticles (MNPs) with NaBH_4_ as a reducing agent for the removal of pollutants from water.^8–12^

The MNPs are more active than their particulate metal counterparts due to their properties, small sizes and large surface areas. Among MNPs, copper nanoparticles (Cu NPs) have attained a particular attention because its cheapness and availability compared to other metal nanoparticles.^13–15^ Despite the advantages of Cu NPs, their agglomeration is inevitable. The synthesis of heterogeneous catalysts is one of the best ways to overcome the agglomeration of MNPs.^16–20^ Recently, various research groups reported the green synthesis of MNPs supported on the various supports such as TiO_2_,^21^ graphene oxide,^22^ Fe_3_O_4_,^23^ bentonite^24^, perlite^25^ and *etc.* Among heterogeneous catalysts, MgO has been used widely as an efficient support and catalyst in organic reactions due to good chemical and thermal stability, low cost, high surface area, ease of handling and high catalytic activity reusability.^26^

Nowadays various techniques have been proposed for the green synthesis of metal nanoparticles using plant extracts as biological materials under mild conditions.^27–30^ Plants extract mediated synthesis of NPs can be beneficial for preparing nanometals to control the size, shape and distribution size. The green approach for the synthesis of MNPs by using plants extract are very desirable compared to other physical and chemical methods because their advantages such as use of nontoxic solvents, simple work-up procedure, very mild reaction conditions, cleaner reaction profiles, elimination of toxic and dangerous materials, elimination of high pressure, energy, temperature, cost effectiveness and dangerous materials without using of surfactant, capping agent and or template.^31–33^

**Fig. 1:**
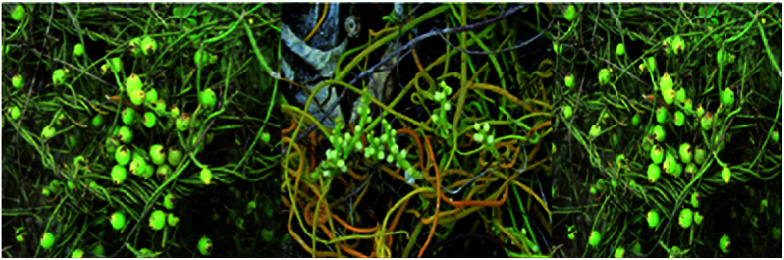
Image of *Cassytha filiformis* L. plant.

*Cassytha filiformis* L. from the Cassythaceae family is a medicinal plant of tropical region of Asia and America which mainly uses in traditional medicine for treatment of cancer, Fig. 1. Because of the presence of its rich phytochemical content in modern medical research, *C. filiformis* has been investigated to possess a number of biologically active chemical compounds with the therapeutic potential in human health applications.^34,35^ Some of the isolated compounds from the extracts of this plant are aporphine alkaloid, oxo-aporphine alkaloid, cassyformine, filiformine, cathaformine, lignan, actinodophine, and octenine. Previous studies on the plant extract strongly support the presence of bioactive phytochemicals such as alkaloids, phenolics, flavonoids, glycosides, resins, proteins, carbohydrates, saponines and tannins.^36–39^ Through this research the aqueous extract of the *Cassytha filiformis* L. was used as reducing media to biosynthesis of stable nanostructures without application of poisonous chemicals and harsh reaction conditions.

In this work, we reported the preparation of Cu/MgO nanocomposite *via* a new, fast, simple, green, cost effective and eco-friendly process by using *Cassytha filiformis* L. extract as stabilizing and reducing agent for the reduction of Cu^2+^ ion to Cu^0^. Then, the catalytic activity of Cu/MgO nanocomposite was investigated against organic dyes such as CR, MB and nitro compounds such as 2,4-DNPH and 4-NP using NaBH_4_ as the source of hydrogen.

## Experimental

### Reagents and methods

All materials with commercial reagent grade were obtained from the Merck and Aldrich companies and used without further purification. FT-IR spectra were recorded on a Nicolet 370 FT/IR spectrometer (Thermo Nicolet, USA) using pressed KBr pellets. The formation of nanoparticles was recorded by UV-visible spectral analysis on a double-beam spectrophotometer (Hitachi, U-2900). The shape and size of the Cu/MgO nanocomposite were identified by transmission electron microscope (TEM) using a Philips EM208 microscope operating at an accelerating voltage of 90 kV. Field emission scanning electron microscopy (FE-SEM) was performed on a cam scan MV2300. EDS (energy dispersive X-ray spectroscopy) was utilized for chemical analysis of prepared nanostructures. X-ray diffraction (XRD) mensuration were carried out using a Philips powder diffractometer type PW 1373 goniometer (Cu Kα = 1.5406 Å). The scanning rate was 2° min^−1^ in the 2*θ* range from 10 to 90°.

### Preparation of *Cassytha filiformis* L. fruit extract

50 g of dried powder of the plant fruit was added to 250 mL double distillated water in 500 mL flask and well mixed. The preparation of extract was using magnetic heating stirrer at 70 °C for 30 min. The obtained extract was centrifuged in 7000 rpm and filtered then filtrate was kept at refrigerator to use further.

### Green synthesis of Cu NPs

**Scheme 1:**
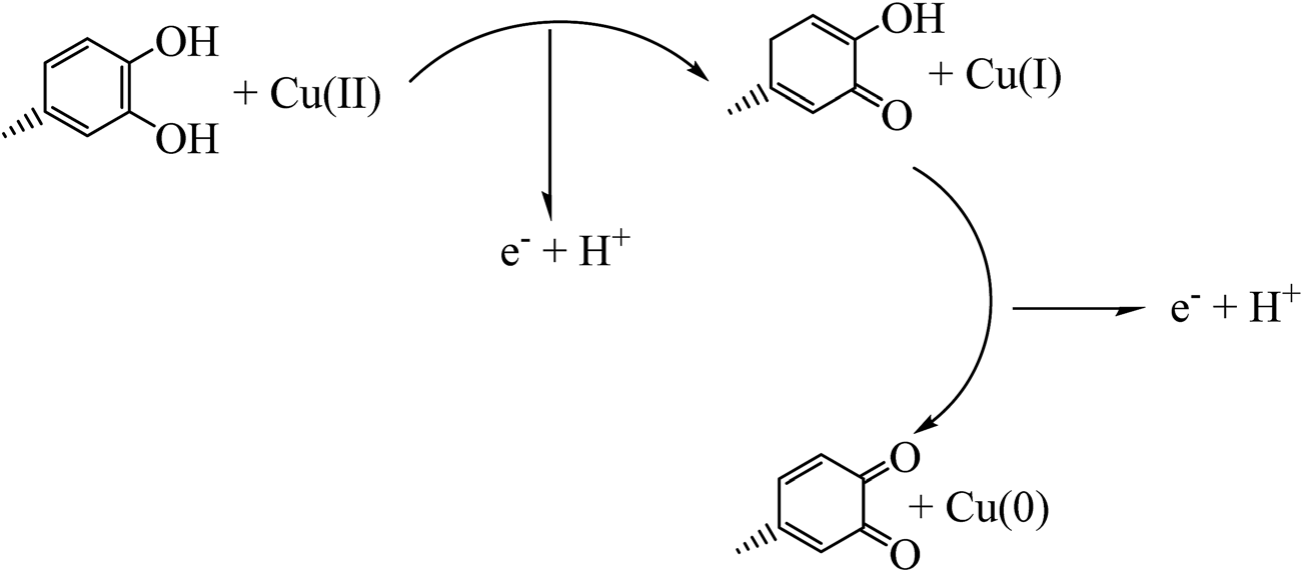
Proposed mechanism for green synthesis of Cu NPs.

In a 250 mL conical flask, 10 mL solution of CuCl_2_·2H_2_O (5 mM) was mixed with 100 mL of the aqueous plant extract along with vigorous shaking until gradually changing the color of the mixture from yellow to dark during 5 min indicating the formation of Cu NPs (as monitored by UV-vis spectrum). The mixture then filtered and centrifuged at 7000 rpm for 30 min and obtained precipitation washed with *n*-hexane and absolute ethanol to remove possible impurities, Scheme 1.

### Preparation of the Cu/MgO nanocomposite using the aqueous extract of the *Cassytha filiformis* L.

For green synthesis of Cu NPs immobilized on MgO as a support, 25 mL of CuCl_2_·2H_2_O (5 mM) was added dropwise to a well-mixed solution of the above extract and 1.0 g of MgO with constant stirring at 80 °C for 4 h. Finally, the prepared Cu/MgO nanocomposite was separated by centrifugation, washed several times with distilled water and then dried at 90 °C for 2 h.

### General procedure for the reduction of 4-NP at room temperature

To evaluate the catalytic activity, a mixture containing 10.0 mg of Cu/MgO nanocomposite and 25 mL of 4-NP aqueous solution (2.5 mM) was stirred for 3 min in a beaker. In the next step, freshly prepared NaBH_4_ aqueous solution (0.25 M, 25 mL) was added and stirred for 5 min at room temperature. The concentration of 4-NP was determined using a Hitachi, U-2900 spectrophotometer. After completion of the reaction, the catalyst was simply separated by brief centrifugation and washed successively with distilled water, dried and used for successive cycles.

### General procedure for the reduction of 2,4-DNPH at room temperature

In a typical experiment, freshly prepared aqueous NaBH_4_ solution (7.91 mM, 25 mL) was added to an aqueous solution that contained 2,4-DNPH (10.076 mM, 25 mL) and 10.0 mg of the Cu/MgO nanocomposite and stirred at room temperature and the reduction process was monitored by recording UV-vis spectra. After completion of the reaction process, the catalyst was filtered, washed with doubly distilled water and then reused.

### General procedure for the reduction of MB and CR at room temperature

In a typical reduction protocol, 25 mL of organic dye solution (MB: 3.1 × 10^−5^ M, CR: 1.44 × 10^−5^ M) was mixed with 10.0 mg of the Cu/MgO nanocomposite and the mixture was stirred at room temperature. Then a freshly prepared NaBH_4_ aqueous solution (5.3 × 10^−3^ M, 25 mL) was added and mixture was stirred at room temperature. The reaction was monitored using the UV-vis spectroscopy. At the end of the reaction, the catalyst was simply separated from the reaction system by brief centrifugation and washed with doubly distilled water and then dried at 100 °C for 2 h for the next cycle.

## Results and discussion

### Preparation and characterization of the Cu/MgO nanocomposite

**Fig. 2:**
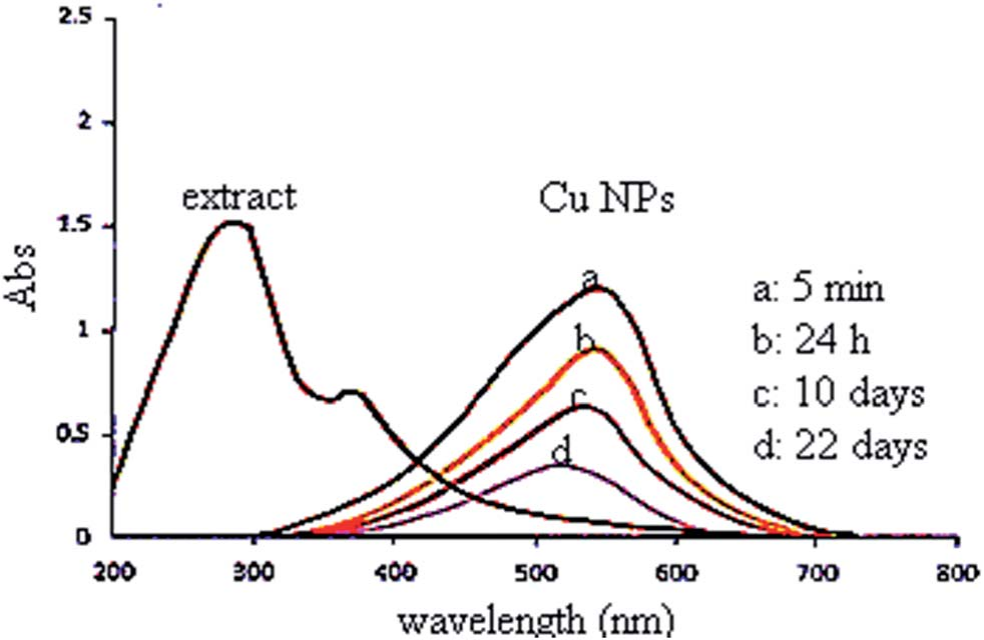
UV-vis spectra of the plant extract and green synthesized Cu NPs.

In this study, the plant extract was used as a reducing and stabilizing agent for the synthesis of Cu NPs without addition of any other external reducing agent. Moreover, the UV spectrum of extract (Fig. 2) shows bands at 375 nm (band I) and 288 nm (band II) assigned to the cinnamoyl and benzoyl systems of phenolics compounds. Therefore, the UV results support the presence of phenolics in plant extract as reported by literatures.^36–39^

The UV-vis spectrum of green synthesized Cu NPs using the plant extract (Fig. 2) showed the significant changes in the absorbance maxima due to surface plasmon resonance. The color of the mixture changed into dark after 5 min at 555 nm indicating formation of Cu NPs as characterized by UV-vis spectrum. The synthesized Cu NPs by this method are quite stable with no significant variance in the shape, position and symmetry of the absorption peak even after 22 days.

**Fig. 3:**
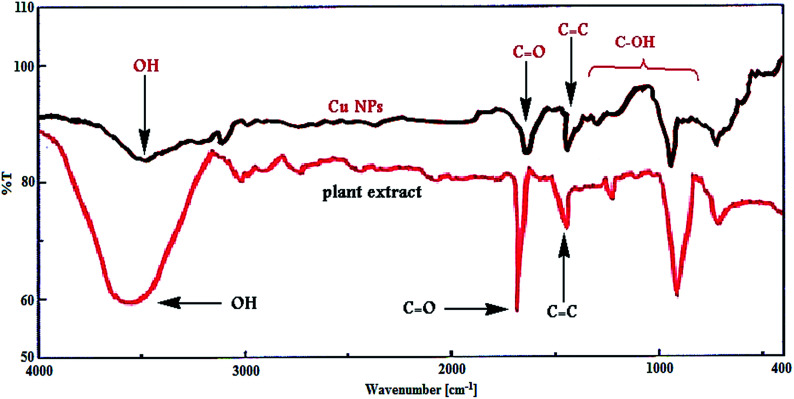
FT-IR spectra of biosynthesized Cu NPs and plant extract.

Furthermore, Fig. 3 shows the FT-IR spectrum of Cu NPs and plant extract for comparison in which shows the interaction between CuCl_2_·2H_2_O and involved sites of phytochemicals to synthesis of Cu NPs (Fig. 3). The peaks at 3500, 1695, 1432, 1300 and 1000 cm^−1^ represent the OH functional groups, carbonyl group (C=O), stretching C=C aromatic ring and C–OH stretching vibrations, respectively. Phytochemicals could adsorb on the surface of metal nanoparticles, possibly by interaction through π-electrons interaction in the absence of other strong ligating agents.

**Fig. 4:**
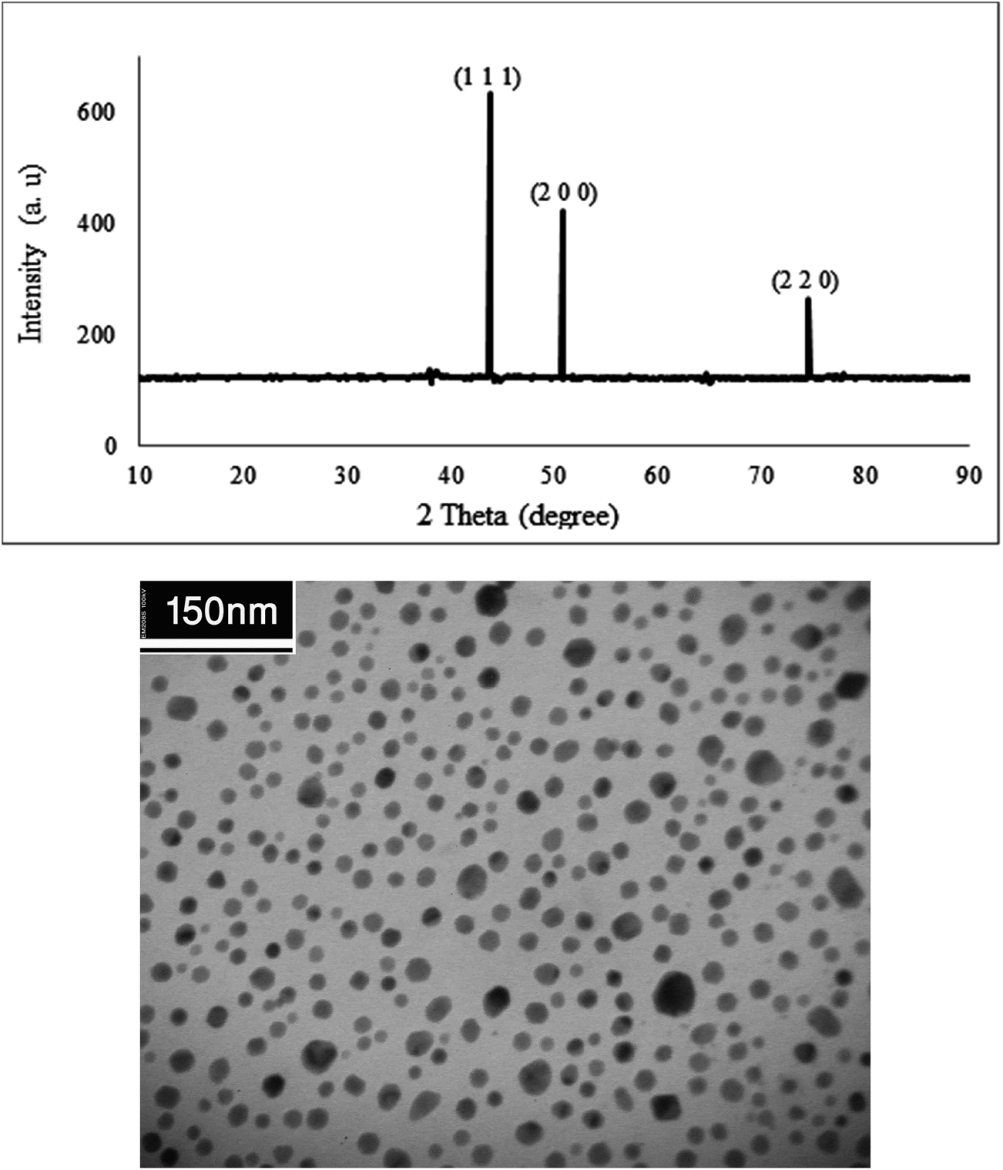
The XRD pattern and TEM image of biosynthesized Cu NPs.

Fig. 4 shows the X-ray diffraction pattern and TEM analysis of the Cu NPs. The patterns at 2*θ* values 43.7°, 50.7° and 74.5° can be assigned to (1 1 1), (2 0 0) and (2 2 0) crystal planes in Cu cubic structure which agrees with the standard Cu (JCPDS 71-4610).

The biosynthesized Cu NPs structure and size was examined using TEM analysis. It is clear from Fig. 4 that the sizes of the Cu NPs are narrow, and the particles are mainly spherical in shape.

The stable Cu/MgO nanocomposite obtained was fully characterized by UV-vis, FT-IR, TEM, SEM and EDS.

**Fig. 5:**
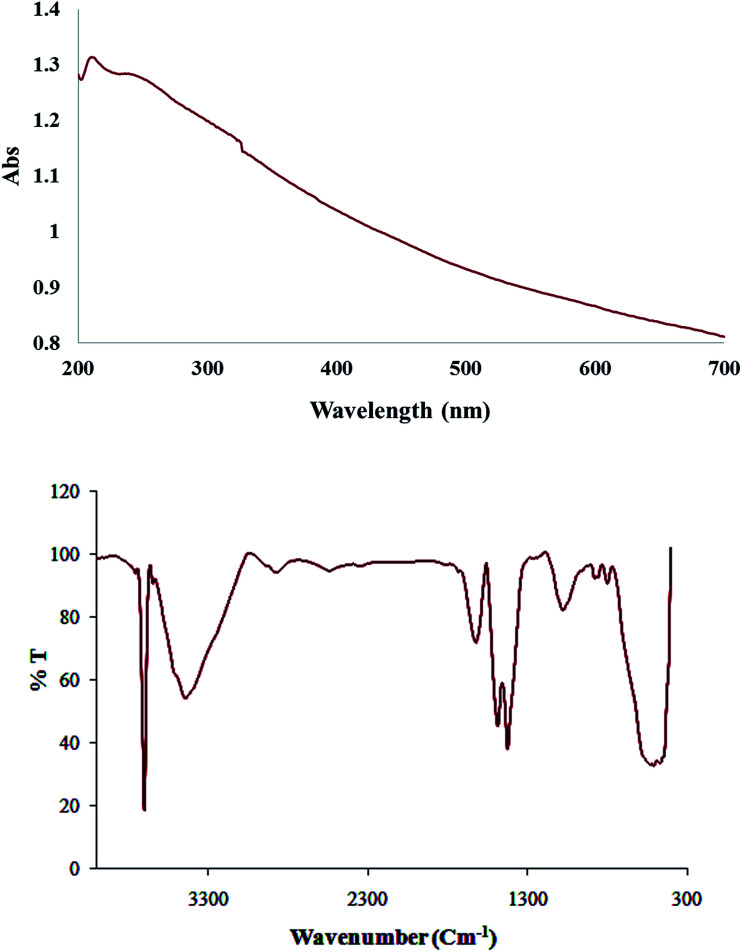
UV-vis and FT-IR spectra of biosynthesized Cu/MgO nanocomposite.

Fig. 5 shows the absorption spectrum of Cu/MgO nanocomposite due to surface plasmon resonance (SPR) of metallic nanoparticles. At compare with spectra related to Cu NPs, changing the color of the reaction (whitish to dark) and decrease the maxima ranging 500–580 nm indicates the reduction process and formation of nanoparticles. As monitored by UV-vis the synthesized nanoparticles by this method are quite stable with no significant variance in the shape, position and symmetry of the absorption peak even after 30 days which indicates the stability of product.

Furthermore, FT-IR spectrum of Cu/MgO nanocomposite is shown in Fig. 5. The appeared bands are lattice vibration modes indicating the functional groups of sample. The bands below 1000 cm^−1^ are related to the Mg-O absorption. A high absorption band, which appeared at 1610 cm^−1^, is also observed in the MgO spectrum, which is related to bending vibration of absorbed water and surface hydroxyl (OH). The peak at 1485 cm^−1^ is assigned to the bending vibration of OH bond. The sharp absorption peak at 3697 cm^−1^ is due to the antisymmetric stretching vibration in the Mg(OH)_2_.

**Fig. 6:**
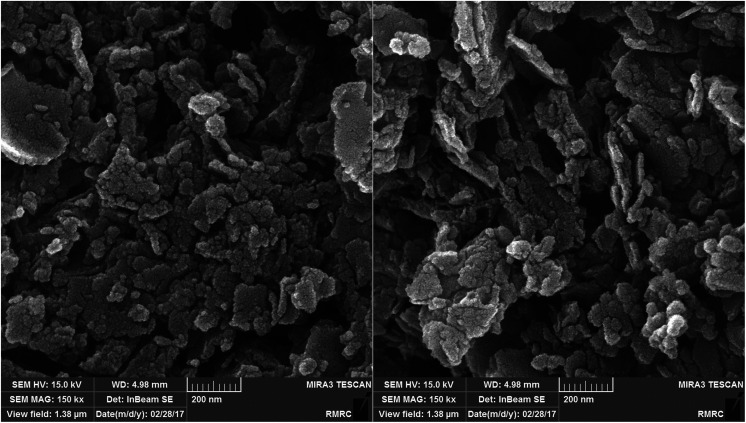
FESEM images of the Cu/MgO nanocomposite.

The morphology of the Cu/MgO nanocomposite is revealed by FESEM. FESEM analysis (Fig. 6) of the Cu/MgO nanocomposite prepared by *Cassytha filiformis* L. showed that copper particles deposited on the rough surface of MgO with flake- and sheet-shaped morphology.

**Fig. 7:**
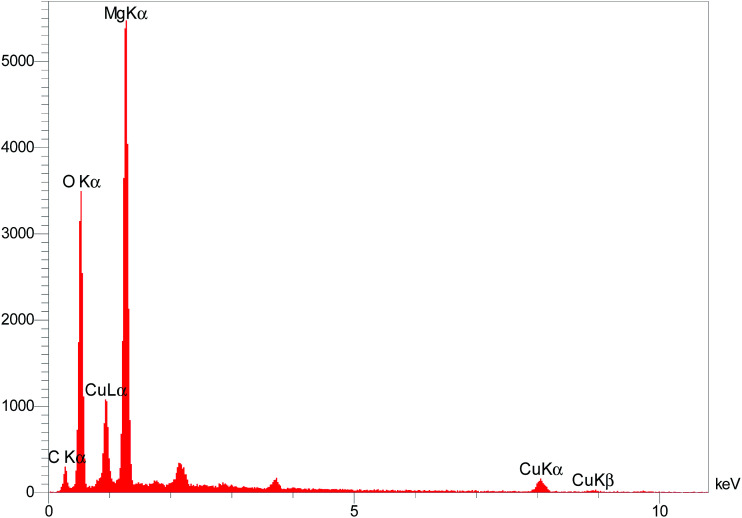
EDS spectrum of the Cu/MgO nanocomposite.

The elemental composition of the Cu/MgO nanocomposite was determined by EDS analysis (Fig. 7). Presence of magnesium (Mg), oxygen (O) and copper (Cu) was confirmed by EDS spectroscopy.

**Fig. 8:**
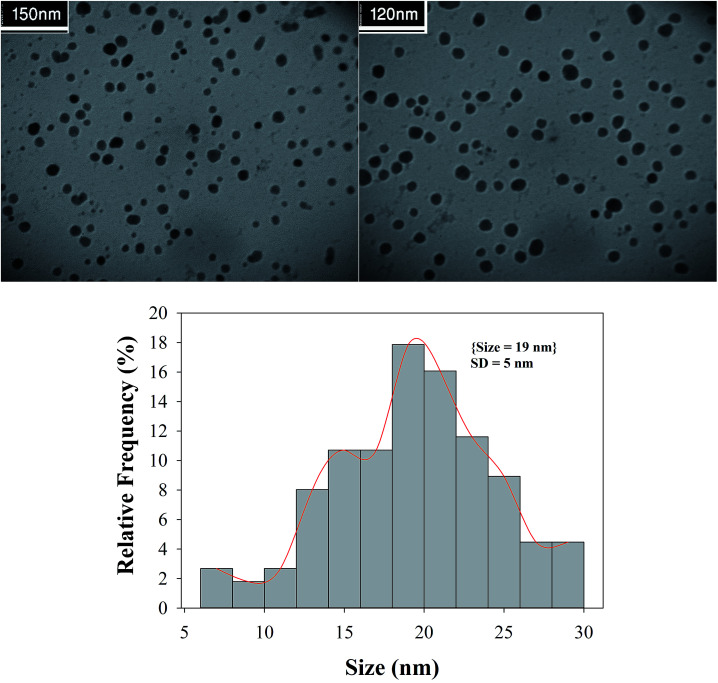
TEM images and histogram of particle size distribution of the Cu/MgO nanocomposite.

A close examination of the TEM images in Fig. 8 reveals that the nanoparticles are spherical morphology in shape. As may be seen in the TEM images, the average diameter of particles is 19 nm.

### Catalytic ability of Cu/MgO nanocomposite for reduction of 4-NP, 2,4-DNPH, MB and CR

Catalytic ability and application of the synthesized Cu/MgO nanocomposite investigated in the reduction of 2,4-DNPH, 4-NP, CR and MB. In the present method, the catalytic reduction of nitro compounds and organic dyes in the presence of NaBH_4_ in water at room temperature was chosen as a pattern reaction to evaluate the catalytic activity of Cu/MgO nanocomposite.

### Catalytic ability of Cu/MgO nanocomposite for reduction of 4-NP at room temperature

**Scheme 2:**
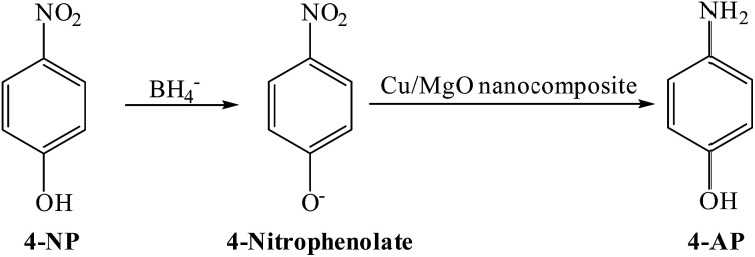
The catalytic reduction of 4-NP to 4-AP at room temperature.

In present work, the catalytic activity of the Cu/MgO nanocomposite was evaluated by the reduction of 4-NP as hazardous matter to 4-AP in the presence of NaBH_4_ as reducing agent in water (Scheme 2).

**Fig. 9:**
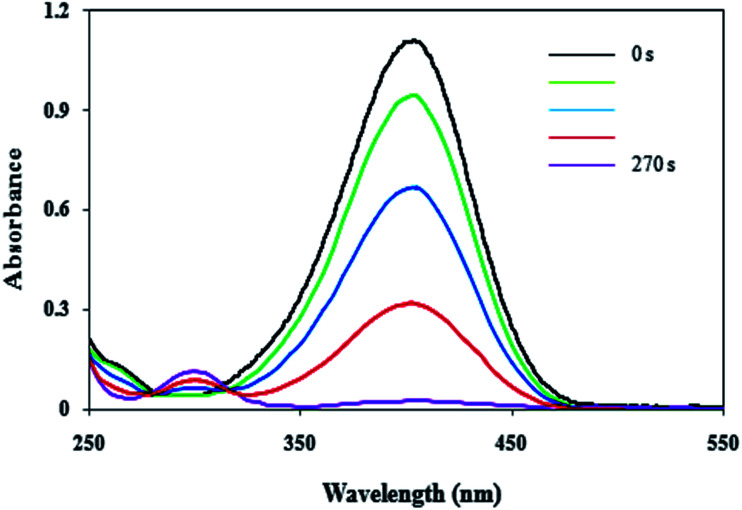
UV-visible spectra of the 4-NP reduced by the Cu/MgO nanocomposite. Reaction conditions: 10.0 mg of Cu/MgO nanocomposite, 25 mL of 4-NP aqueous solution (2.5 mM) and 25 mL of NaBH_4_ aqueous solution (2.5 mM) at room temperature.

4-NP in aqueous medium has a maximum absorption at 317. After the addition of the NaBH_4_ solution, the new absorption peak at 400 nm is appeared due to formation of 4-nitrophenolate (Fig. 9). In the presence of 4-NP + NaBH_4_ solution and in the absence of Cu/MgO nanocomposite does not happen any reduction process under alkaline conditions (Table 1, entry 1). As show in Fig. 9, after the addition of the Cu/MgO nanocomposite to 4-NP + NaBH_4_ solution, a new absorption signal at 300 nm is appeared following the formation of 4-AP and the solution color changes from yellow to colorless. We also investigated the effect of the amount of the NaBH_4_ for the reduction of 4-NP. In the absence of NaBH_4_, no reduction occurred within the reaction time (entry 3 and 9). The best result was obtained with 10.0 mg of Cu/MgO nanocomposite with 100 equivalents of NaBH_4_ (Table 1, entry 4). After completion of the reaction and conversion of 4-NP to 4-AP, the peak at 400 nm was disappeared. As shown in Table 1, the reaction took place after 520 s in the presence of Cu NPs. According the results, Cu/MgO nanocomposite is much more reactive than Cu NPs and the reaction was completed during 270 s in the presence of the Cu/MgO nanocomposite. The MgO is not only a support to prevent aggregation of the Cu NPs, but also can provide a synergistic effect in reduction process, which allow more molecules to be in contact with the surface of Cu NPs.

**Table 1:** Completion time for the reduction 4-NP to 4-AP at room temperature using different amounts of Cu/MgO nanocomposite

Entry	Catalyst (mg)	NaBH_4_ (equivalents)	Time (min)
1	—	100	140 min^a^
2	MgO (10.0)	100	40 min^b^
3	Cu/MgO nanocomposite (7.0)	0.0	300 min^b^
4	Cu/MgO nanocomposite (10.0)	100	270 s
5	Cu/MgO nanocomposite (7.0)	100	280 s
6	Cu/MgO nanocomposite (5.0)	100	537 s
7	Cu/MgO nanocomposite (10.0)	79	477 s
8	Cu/MgO nanocomposite (10.0)	50	10 min
9	Cu NPs	0.0	300 min^b^
10	Cu NPs	100	520 s

a No reaction.

b Not completed.

The reduction process of 4-NP to 4-AP is usually carried out in presence of a catalyst in aqueous media at room temperature. Therefore, presence of the Cu/MgO nanocomposite as a catalyst and NaBH_4_ as a reducing agent is necessary for reduction of 4-NP. In the absence of the Cu/MgO nanocomposite and NaBH_4_, reduction reaction did not proceed even after a long time (Table 1).

**Scheme 3:**
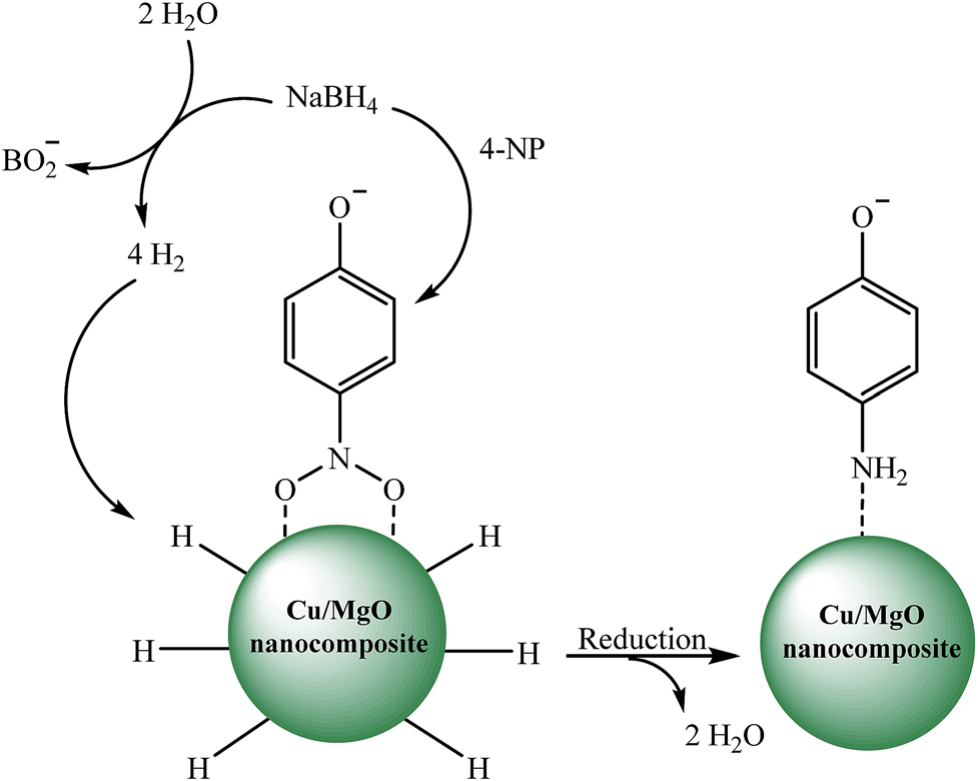
Possible mechanism for the catalytic reduction of 4-NP to 4-AP by Cu/MgO nanocomposite at room temperature.

The catalytic reduction of the 4-NP by using Cu/MgO nanocomposite is an electron transfer (ET) process. The reaction was carried out in two steps (Scheme 3). In the first step of process, 4-NP and BH_4_ diffuse from aqueous solution to the surface of catalyst *via* π–π stacking interactions. In the next step, after electron transfer from the BH_4_^−^ (reductant) and 4-NP (oxidant) near to the Cu NPs on the MgO surface as the electron mediator, the hydrogen atoms, which are formed from BH_4_^−^, attack 4-NP molecule to reduce it. Finally, the corresponding product was desorbed from the surface of the catalyst. These observations indicate that the MgO can stabilize the supported Cu NPs against aggregation also enhance the catalytic activity through a synergistic effect.

### Catalytic ability of Cu/MgO nanocomposite for reduction of 2,4-DNPH at room temperature

**Scheme 4:**
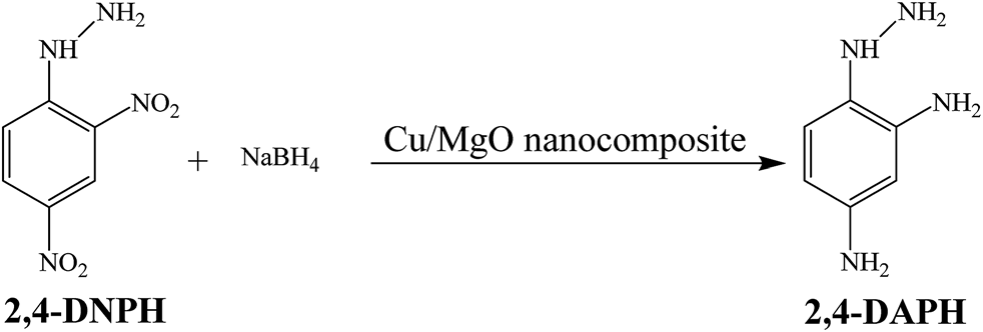
The catalytic reduction of 2,4-DNPH to 2,4-DAPH in aqueous medium at room temperature.

The catalytic reduction of 2,4-DNPH aqueous solution with NaBH_4_ at room temperature has also been used as another model reaction to check the catalytic activity of the Cu/MgO nanocomposite (Scheme 4).

**Fig. 10:**
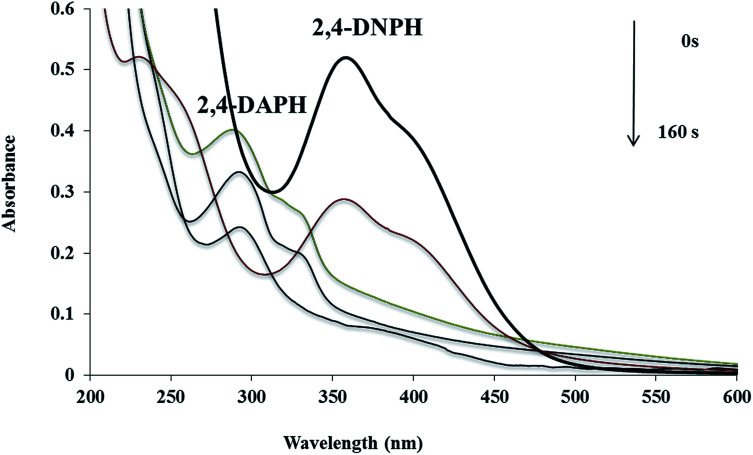
UV-visible spectra of the 2,4-DNPH reduced by the Cu/MgO nanocomposite. Reaction conditions: 10.0 mg of Cu/MgO nanocomposite, 25 mL of 2,4-DNPH (10.076 mM) and 25 mL of NaBH_4_ solution (7.91 mM) at room temperature.

The Cu/MgO nanocomposite demonstrated high activity for reduction of 2,4-DNPH to 2,4-DAPH in very short time (Table 2). This process was monitored by UV-vis spectroscopy. The 2,4-DNPH in aqueous medium has a maximum absorption at 353 nm (Fig. 10). After the addition of NaBH_4_ to 2,4-DNPH solution in the absence of the Cu/MgO nanocomposite no change in the color of the solution and absorption spectrum was observed even after 120 min (Table 2, entry 1). After the addition of Cu/MgO nanocomposite to the solution, a new absorption signal at 290 nm is appeared due to the formation of 2,4-DAPH. The effect of the concentration of NaBH_4_ and catalyst loading was studied by carrying out the reduction reaction in the presence of varying amounts of the NaBH_4_ and catalyst. In the absence of NaBH_4_, no reduction occurred within the reaction time. The best result was obtained with 104 equivalents of NaBH_4_ and 10.0 mg of the Cu/MgO nanocomposite (Table 2, entry 8).

**Table 2:** Completion time for the reduction 2,4-DNPH to 2,4-DAPH at room temperature using different amounts of Cu/MgO nanocomposite and NaBH_4_

Entry	Catalyst (mg)	NaBH_4_ (equivalents)	Time
1	—	104	120 min^a^
2	MgO (10.0)	104	7 min
3	Cu/MgO nanocomposite (5.0)	75	25 min^b^
4	Cu/MgO nanocomposite (5.0)	104	5 min
5	Cu/MgO nanocomposite (7.0)	104	220 s
6	Cu/MgO nanocomposite (10.0)	50	15 min^b^
7	Cu/MgO nanocomposite (10.0)	75	15 min^b^
8	Cu/MgO nanocomposite (10.0)	104	160 s

a No reaction.

b Not completed.

### Catalytic ability of the Cu/MgO nanocomposite for reduction of the MB and CR at room temperature

**Fig. 11:**
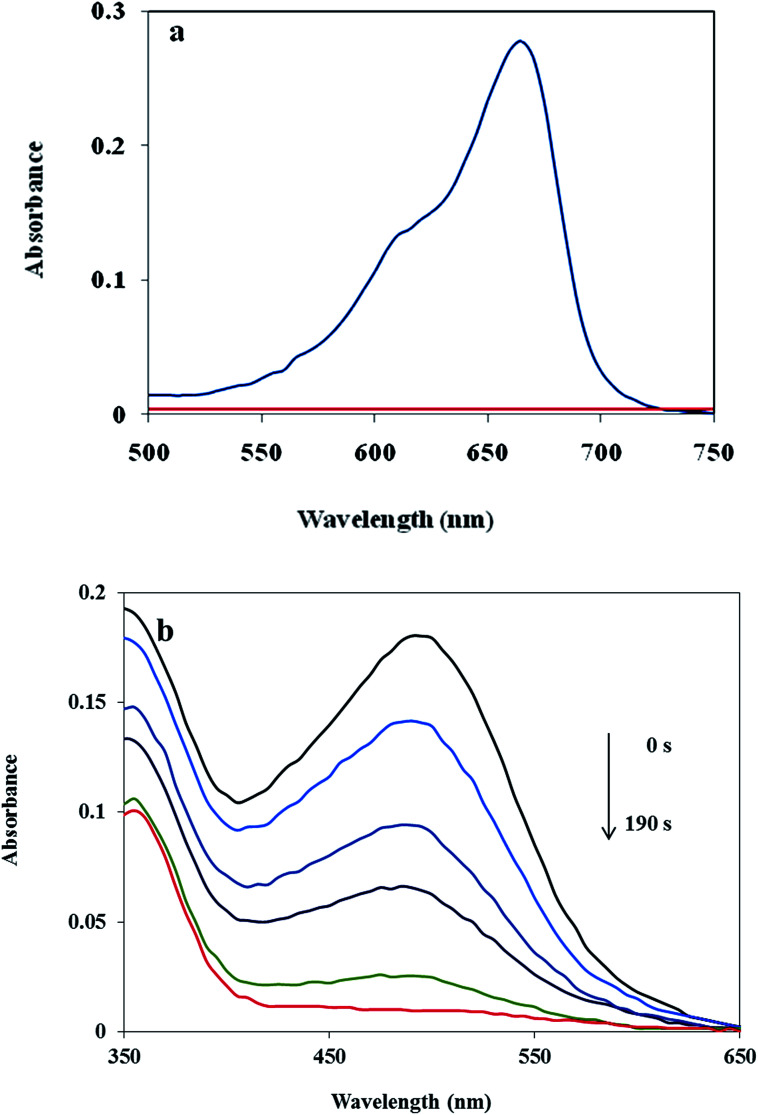
UV-visible spectra of the MB (a) and CR (b) reduced by using Cu/MgO nanocomposite at room temperature.

In addition, the reduction of the MB and CR were chosen to evaluate the performance of the as-prepared Cu/MgO nanocomposite. The catalytic reduction of MB and CR was investigated with 25 mL of fresh NaBH_4_ aqueous solution (5.3 × 10^−3^ M) and different amounts of catalyst at room temperature. The MB and CR dyes represented a characteristic SPR band at *λ*_max_ 663 and 493 nm, respectively (Fig. 11). It is mentioned here that the no decolorization of dye could be detected in the absence of the NaBH_4_ or catalyst. The effect of the catalyst loading is a significant issue for the reduction of dyes. As shown in Table 3, it was observed that reduction of MB and CR occurred within 1 s and 190 s, respectively in the presence of 10.0 mg of the Cu/MgO nanocomposite. The progress of the reaction was monitored using UV-visible measurements at ordered intervals of time. The catalytic activity of the prepared Cu NPs by *Cassytha filiformis* L. extract was also tested for the reduction of MB and CR (Table 3, entry 4 and 8). It is interesting to note that the Cu NPs gave poorer activity.

**Table 3:** Optimization of reaction conditions for reduction of the MB and CR

Entry	Dye (M)	Catalyst (mg)	Time (s)
1	MB (3.1 × 10^−5^)	Cu/MgO nanocomposite (5.0)	6
2	MB (3.1 × 10^−5^)	Cu/MgO nanocomposite (7.0)	3
3	MB (3.1 × 10^−5^)	Cu/MgO nanocomposite (10.0)	1
4	MB (3.1 × 10^−5^)	Cu NPs (10.0)	17
5	CR (1.44 × 10^−5^)	Cu/MgO nanocomposite (5.0)	300
6	CR (1.44 × 10^−5^)	Cu/MgO nanocomposite (7.0)	256
7	CR (1.44 × 10^−5^)	Cu/MgO nanocomposite (10.0)	190
8	CR (1.44 × 10^−5^)	Cu NPs (10.0)	410

The catalytic activity of the prepared Cu/MgO nanocomposite towards 2,4-DNPH, 4-NP, MB and CR reduction was compared with reported catalysts in the literature. According to Table 4, the Cu/MgO nanocomposite exhibited a higher catalytic activity than other catalysts. As shown in Table 4, the best result was obtained with Cu/MgO nanocomposite at room temperature with shorter reaction times. In addition, the Cu/MgO nanocomposite were synthesized by a green technique using *Cassytha filiformis* L. extract without use of dangerous and toxic reagents or capping agent and surfactant.

**Table 4:** Data for the catalytic reduction of 4-NP, 2,4-DNPH, CR and MB in presence of NaBH_4_ by different catalysts

Substrate	Catalyst	Concentration (mL, mM)	NaBH_4_ (mL, mM)	Time (min)	Ref.
4-NP	Au@PZS@CNTs (0.3 mL, 1 mg mL^−1^)	1.7, 0.2	1.0, 15.0	16	40
	Au–GO (0.25 mL, 1.4 × 10^−4^ M)	10, 0.750	1.0, 2.22 × 10^−3^	7	41
	Ni NPs (3.0 mg)	3.0, 0.1	0.3, 200.0	16	42
	Cu microspheres (0.5 mg)	30.0, 0.2	10.0, 25.0	18	43
	Pd–FG (1.0 mg)	2.9, 0.1	0.1, 10.0	12	44
	Cu/MgO nanocomposite (10.0 mg)	25, 2.5	25.0, 250.0	4	This work
2,4-DNPH	Ag@AgCl NPs (150 μL, (0.008 g))	60.0 μL, 6.0	350.0 μL, 0.1 M	60	45
	Cu/MgO nanocomposite (10.0 mg)	25.0, 10.076	25.0, 7.91	150 s	This work
CR	Cu@SBA-15 (1.0 mg)	22.5, 0.09	5.0, 200.0	7	46
	Cu/MgO nanocomposite (10.0 mg)	25.0, 1.44 × 10^−2^	25.0, 5.3	130 s	This work
MB	Au@TiO_2_ (2.0 mg)	20.0, 0.04	2.0, 100.0	12	47
	Natrolite zeolite/Pd (7.0 mg)	25.0, 0.03	25.0, 5.3	1 s	48
	Cu/MgO nanocomposite (10.0 mg)	25.0, 3.1 × 10^−2^	25.0, 5.3	1 s	This work

### Catalyst recyclability

**Fig. 12:**
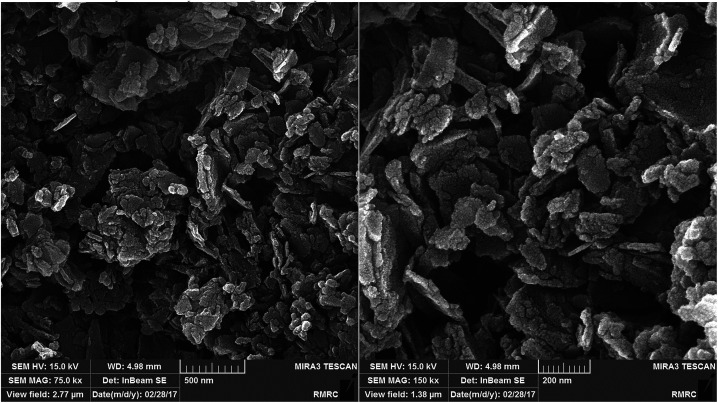
FESEM images of recovered Cu/MgO nanocomposite.

**Fig. 13:**
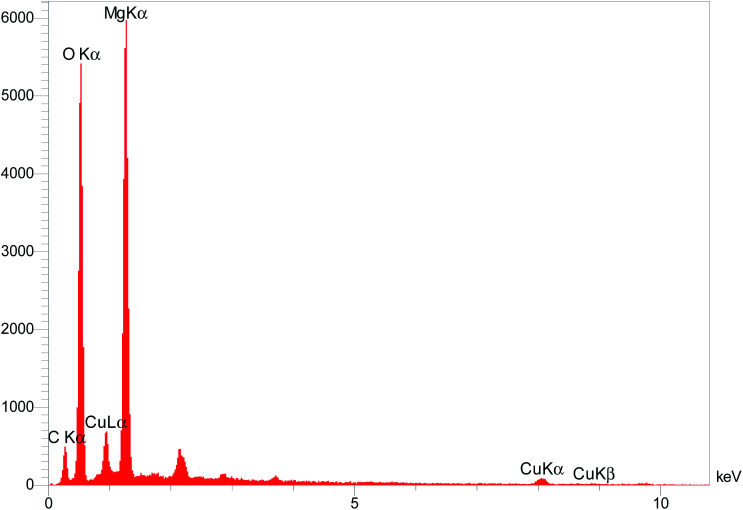
EDS analysis of recovered Cu/MgO nanocomposite.

**Fig. 14:**
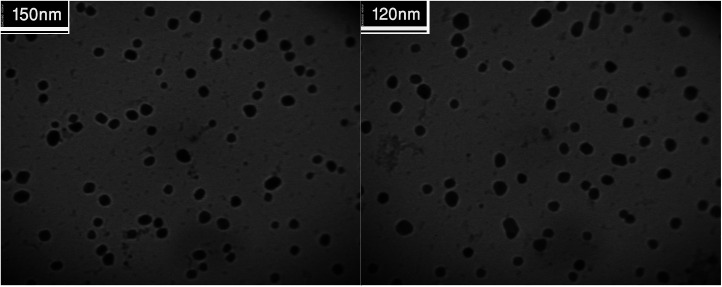
TEM images of recovered Cu/MgO nanocomposite.

**Fig. 15:**
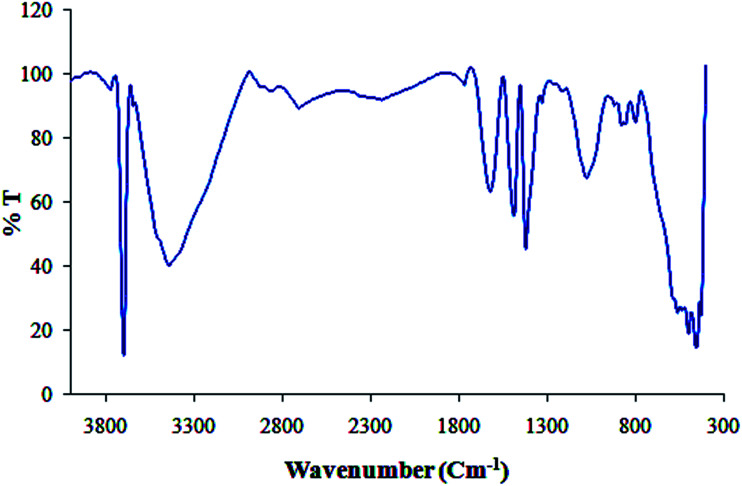
FT-IR spectrum of recovered Cu/MgO nanocomposite.

The recyclability of the catalysts is one of the advantages of heterogeneous catalysts. The stability and reusability of the Cu/MgO nanocomposite was tested in the reduction of CR with NaBH_4_. The Cu/MgO nanocomposite can be separated from the reaction mixture by mild centrifugation and washed with distilled water several times, dried and then reused at least six times without significant loss of catalytic activity. The high activity of catalyst confirms the high stability of Cu/MgO nanocomposite under the reaction conditions. As shown in TEM, FESEM, FT-IR images and EDS analysis of the recycled catalyst (Fig. 12–15), no obvious change in structure, chemical composition, morphology and size of NPs were observed. The XRD patterns before and after the reaction revealed that the Cu/MgO nanocomposite retained its crystallinity throughout.

## Conclusion

The green synthesis of copper nanoparticles using *Cassytha filiformis* L. extract provides a rapid and simple route for the preparation of Cu/MgO nanocomposite. The flavonoids present in extract of *Cassytha filiformis* L. act as both reducing and capping/stabilizing agents. The synthesized Cu and Cu/MgO nanocomposite were characterized by XRD, SEM, EDS, TEM, FT-IR and UV-vis spectroscopic techniques. The catalyst exhibits high catalytic activity for the reduction of 2,4-DNPH, 4-NP, MB and CR by using NaBH_4_ in aqueous medium at room temperature. The significant advantages of this methodology are elimination of hazardous materials, short reaction time, mild reaction conditions and simple work-up procedure.

## Conflicts of interest

There are no conflicts to declare.

## Supplementary Material
